# A weight regain of 1.5 kg or more and lack of exercise are associated with nonalcoholic fatty liver disease recurrence in men

**DOI:** 10.1038/s41598-021-99036-y

**Published:** 2021-10-07

**Authors:** Naoko Nakanishi, Yoshitaka Hashimoto, Takuro Okamura, Akihiro Ohbora, Takao Kojima, Masahide Hamaguchi, Michiaki Fukui

**Affiliations:** 1 Department of Endocrinology and Metabolism, Graduate School of Medical Science, Kyoto Prefectural University of Medicine, 465, Kajii-cho, Kawaramachi-Hirokoji, Kamigyo-ku, Kyoto, 602-8566 Japan; 2 Department of Gastroenterology, Asahi University Hospital, Gifu, Japan

**Keywords:** Non-alcoholic fatty liver disease, Epidemiology

## Abstract

The importance of maintaining the remission of nonalcoholic fatty liver disease (NAFLD) has been overlooked. Here we aimed to clarify factors causing NAFLD recurrence. In this retrospective cohort study over 10.8 ± 5.4 years, we investigated 1260 male health check-up participants diagnosed with NAFLD who achieved remission. The data were compared between the maintained remission and recurrence group. Among all participants, 618 (49.0%) showed NAFLD recurrence at the last visit. Participants in the maintained remission group continued to lose weight (72.7 ± 9.1, 68.7 ± 8.5 and 68.2 ± 8.9 kg), whereas those in the recurrence group lost and regained weight (72.9 ± 9.9, 69.7 ± 9.3 and 73.0 ± 10.4 kg). Receiver operating characteristic curve analysis showed a weight regain of + 1.5 kg as the cutoff value for recurrence. The proportion of regular exercisers at the last visit was 34.6% in the maintained remission group and 24.5% in the recurrence group (*p* < 0.0001). Multivariable analysis revealed the amount of weight regain (in 1 kg increments; adjusted odds ratio, 1.29; 95% confidence interval, 1.24–1.34) and regular exercise at the last visit (adjusted odds ratio, 0.67; 95% confidence interval, 0.55–0.89) were independently associated with recurrence. These findings demonstrate a weight regain of 1.5 kg or more and lack of exercise were associated with NAFLD recurrence.

## Introduction

The number of patients with nonalcoholic fatty liver disease (NAFLD) is increasing worldwide^1^. Some individuals with NAFLD develop nonalcoholic steatohepatitis (NASH), which causes liver fibrosis and increases their risk of hepatocellular carcinoma^2,3^. NAFLD has mutual and bi-directional association with metabolic syndrome, which includes hypertriglyceridemia, low high-density lipoprotein (HDL) level, and glucose intolerance^4–6^. Further, patients with NAFLD are at an increased risk of developing cardiovascular disease^7,8^. In addition, NAFLD and type 2 diabetes mellitus form a vicious cycle in the progression of both conditions^9,10^. The development of strategies for controlling NAFLD is required to prevent the progression of type 2 diabetes^11^.

NAFLD is reversible to a certain extent. Various reports have shown that weight loss and lifestyle interventions effectively improve NAFLD^12,13^ and NASH^14,15^. The Japan Society of Hepatology recommends a body weight loss of > 7% to improve NAFLD and NASH. With respect to lifestyle modifications, exercise and calorie restriction are crucial for NAFLD improvement^16,17^. However, thus far, most studies have focused on treatments to achieve NAFLD remission. Although a fair number of studies examined the factors contributing to NAFLD recurrence after liver transplantation^18,19^, very few studies have investigated the natural history of patients who achieved remission through lifestyle changes. Thus, the factors that determine whether individuals maintain NAFLD remission or develop recurrence remain unclear.

To answer this question, we designed this retrospective cohort analysis of health check-up participants and examined changes in lifestyle history such as exercise and smoking habits obtained by questionnaires as well as clinical and laboratory data including metabolic syndrome parameters. This study aimed to reveal the factors associated to NAFLD recurrence diagnosed by abdominal ultrasonography. Considering that sex differences do exist in the prevalence and risk factors of NAFLD^20^, we analyzed the data separately by sex.

## Results

We conducted a cohort analysis of participants who underwent a medical health check-up including abdominal ultrasonography. The evaluations were performed in the Medical Health Checkup Center at Asahi University Hospital, Gifu, Japan. We named the longitudinal cohort analysis using this database NAGALA (NAFLD in Gifu Area Longitudinal Analysis).

**Figure 1:**
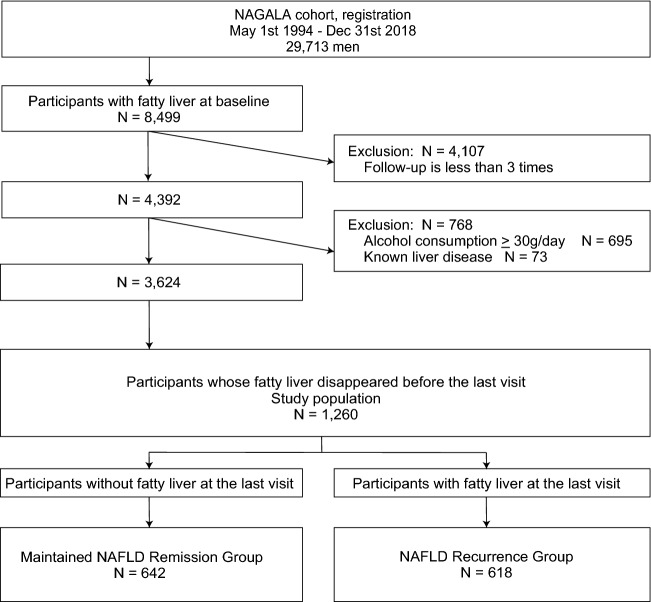
Flow diagram of study population selection in men. NAGALA, NAFLD in Gifu Area, Longitudinal Analysis; NAFLD, nonalcoholic fatty liver disease.

The present cohort study of men is shown in the flow diagram in Fig. 1. From the men who were registered as NAGALA between May 1994 and Dec 2018, we recruited those in whom fatty liver was diagnosed using abdominal ultrasonography at the first visit and who underwent at least three health examinations during the observation period. We excluded participants with an alcohol consumption of ≥ 30 g/day (695 participants) and those who had known liver disease including viral hepatitis (73 participants). Among the remaining 3624 participants, we sorted out the participants who showed disappearance of fatty liver on abdominal ultrasonography before the last visit. The earliest time point when fatty liver disappeared was set as the time of NAFLD remission. Finally, 1260 participants were investigated in the male study.

The data were compared between the maintained NAFLD remission group, in which fatty liver disappeared and was not identified at the last visit; and the NAFLD recurrence group, in which fatty liver once disappeared but reappeared at the last visit. Among these 1260 participants who achieved NAFLD remission during the observation period, 642 (51.0%) remained in remission until the last visit and 618 (49.0%) showed recurrence at the last visit (Fig. 1). We examined the data at three different time points: the first visit, time when fatty liver disappeared (NAFLD remission), and date of last visit.

The mean follow-up duration was 10.8 ± 5.4 years. The follow-up duration in the maintained NAFLD remission group was 10.0 ± 5.6 years and 11.6 ± 5.1 years in the NAFLD recurrence group (*p* < 0.0001).

The clinical and laboratory characteristics at baseline of the whole cohort and each group in men are summarized in Table 1. At baseline, the NAFLD recurrence group had a lower mean age, higher proportion of current smokers, higher mean serum aspartate aminotransferase level, higher mean serum alanine aminotransferase level, lower mean hemoglobin A1c (HbA1c) level, and higher mean serum triglyceride level than the maintained NAFLD remission group. There were no significant intergroup differences in mean body weight, body mass index (BMI), and exercise habits at baseline.

**Table 1:** Baseline characteristics of male study participants in the whole cohort and each group.

Characteristics	ALL n = 1260	Maintained NAFLD remission n = 642	NAFLD recurrence n = 618	*p*
Age (year)	44.7 (8.6)	45.6 (8.9)	43.8 (8.3)	0.0002
Body weight (kg)	72.8 (9.5)	72.7 (9.1)	72.9 (9.9)	0.6602
Body mass index (kg/m^2^)	25.1 (2.7)	25.1 (2.5)	25.2 (2.8)	0.4923
Parental history of diabetes (yes)	91 (7.2)	44 (6.9)	47 (7.6)	0.6064
Waist circumference (cm)	86.3 (6.9)	86.3 (6.5)	86.2 (7.3)	0.7799
Alcohol (yes)	857 (68.0)	446 (69.5)	411 (66.5)	0.2592
Smoking (Non)	408 (32.5)	199 (31.2)	209 (33.8)	
(Past)	418 (33.3)	234 (36.7)	184 (29.8)	
(Current)	430 (34.2)	205 (32.1)	225 (36.4)	0.0327
Regular exercise (yes)	202 (16.1)	104 (16.2)	98 (15.9)	0.8889
Coffee intake (yes)	959 (76.2)	489 (76.3)	470 (76.0)	0.9220
Sleep duration per day (hour)	6.3 (0.9)	6.4 (0.9)	6.3 (0.9)	0.4256
Systolic blood pressure (mmHg)	125.6 (15.3)	126.3 (15.5)	124.8 (15.0)	0.0816
Diastolic blood pressure (mmHg)	79.2 (10.0)	79.7 (10.0)	78.7 (10.1)	0.0755
Aspartate aminotransferase (IU/L)	22.0 (8.4)	21.4 (8.0)	22.5 (8.8)	0.0251
Alanine aminotransferase (IU/L)	32.1 (17.6)	30.4 (16.2)	34.0 (18.8)	0.0003
γ-Glutamyltransferase (IU/L)	26 (19–38)	25 (18–38)	26 (19–38)	0.6109
eGFR (ml/min/1.73 m^2^)	69.1 (12.5)	69.0 (13.1)	69.3 (11.7)	0.7096
Fasting plasma glucose (mmol/L)	5.8 (1.1)	5.8 (1.1)	5.7 (1.1)	0.0989
HbA1c (%)	5.5 (0.8)	5.6 (0.9)	5.5 (0.8)	0.0031
Total cholesterol (mmol/L)	5.5 (0.9)	5.5 (0.9)	5.5 (0.9)	0.6351
Triglycerides (mmol/L)	1.4 (1.0–2.1)	1.4 (1.0–2.0)	1.5 (1.1–2.1)	0.0125
HDL cholesterol (mmol/L)	1.1 (0.3)	1.1 (0.3)	1.1 (0.3)	0.1268
FIB-4 index (≤ 1.30)	1151 (92.0)	589 (92.3)	562 (91.7)	
(> 1.30 and < 2.67)	95 (7.6)	46 (7.2)	49 (8.0)	
(≥ 2.67)	5 (0.4)	3 (0.5)	2 (0.3)	0.8071

**Figure 2:**
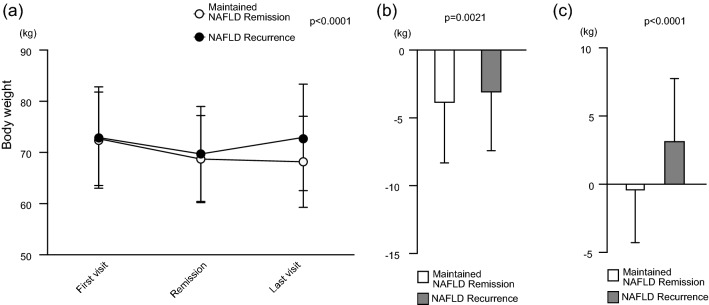
Body weight change in each group during the observation period. Data are shown as mean ± SD. (**a**) Changes in body weight. The p value was calculated using two-way repeated-measures analysis of variance. (**b**) Amount of weight change between the first visit and the date of NAFLD remission. The p value was calculated using Student’s t test. (**c**) Amount of weight change between the date of NAFLD remission and the last visit. The p value was calculated using Student’s t test. NAFLD, nonalcoholic fatty liver disease.

The mean body weight change at the three time points during the observation period is shown in Fig. 2. The participants in the maintained NAFLD remission group continued to lose weight (72.7 ± 9.1, 68.7 ± 8.5, and 68.2 ± 8.9 kg), whereas those in the NAFLD recurrence group lost and subsequently regained weight (72.9 ± 9.9, 69.7 ± 9.3, and 73.0 ± 10.4 kg). Two-way repeated-measures analysis of variance revealed a significant interaction of time and groups on body weight changes (*p* < 0.0001) (Fig. 2a). Between the first visit and the date of NAFLD remission, the mean weight reduction was -4.0 ± 4.5 kg in the maintained NAFLD remission group and -3.2 ± 4.3 kg in the NAFLD recurrence group (*p* = 0.0021) (Fig. 2b). Between the date of NAFLD remission and the last visit, however, a further body weight reduction of -0.5 ± 3.9 kg was observed in the maintained NAFLD remission group, while a weight regain of + 3.2 ± 4.6 kg was observed in the NAFLD recurrence group (*p* < 0.0001) (Fig. 2c).

**Figure 3:**
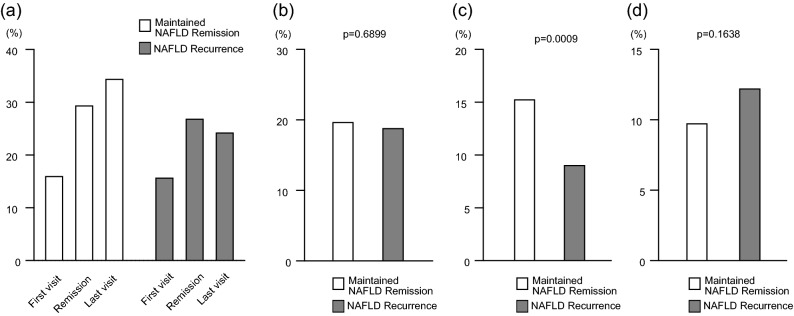
Changes in exercise habits. Values are presented as percentages. The p values were calculated using Pearson’s χ2 test. (**a**) Proportions of regular exercisers during the observation period. (**b**) Proportions of participants who had newly started exercising by the date of NAFLD remission. (**c**) Proportions of participants who newly started exercising after the date of NAFLD remission. (**d**) Proportions of participants who stopped regular exercise after the date of NAFLD remission. NAFLD, nonalcoholic fatty liver disease.

The proportions of regular exercisers during the observation period are shown in Fig. 3a. The proportion of regular exercisers in the maintained NAFLD remission group continued to increase, whereas that in the NAFLD recurrence group increased between the first visit and the date of NAFLD remission and decreased between the date of NAFLD remission and the last visit. The proportion of regular exercisers at the last visit was 34.6% and 24.5% in the maintained remission and recurrence groups, respectively (*p* < 0.0001). No significant difference was observed in the proportion of participants who had newly started exercising by the date of NAFLD remission (19.9% vs. 19.0%, *p* = 0.6899) (Fig. 3b). After the date of NAFLD remission, however, the NAFLD recurrence group had a significantly lower proportion of participants who newly started exercising (15.4% vs. 9.2%, *p* = 0.0009) (Fig. 3c) and a higher proportion of participants who stopped regular exercise (9.8% vs. 12.3%, *p* = 0.1638) (Fig. 3d). The initiation or continuation of regular exercise at least once a week was associated with a lower incidence of NAFLD recurrence.

The proportion of current smokers continued to decrease in both groups. No significant intergroup difference was observed in the proportion of participants who had quit smoking by the date of NAFLD remission (6.8% vs. 7.1%, *p* = 0.8203) However, the proportion of participants who quit smoking was higher in the NAFLD recurrence group than in the maintained NAFLD remission group after NAFLD remission was achieved (5.6% vs. 8.6%, *p* = 0.0398). No significant intergroup differences were observed in alcohol consumption, coffee intake, and sleep duration.

The factors associated with NAFLD recurrence were assessed by univariable and multivariable logistic regression (Table 2). The covariables included the baseline characteristics, body weight change, lifestyle changes, and follow-up period that were significant associated with NAFLD recurrence. The multivariable logistic regression analysis showed that age, follow-up duration, amount of weight change after the date of NAFLD remission (in 1 kg increments; adjusted odds ratio [aOR], 1.29; 95% confidence interval [CI], 1.24–1.34; *p* < 0.0001), and regular exercise at the last visit (aOR, 0.67; 95% CI, 0.55–0.89; *p* = 0.0053) were independently associated with NAFLD recurrence.

**Table 2:** Odds ratio of NAFLD recurrence in men.

	Unadjusted		Adjusted	
	Odds ratio (95% confidence interval)	*p*	Odds ratio (95% confidence interval)	*p*
Age at baseline (year)	0.98 (0.96–0.99)	0.0003	1.02 (1.00–1.04)	0.0234
Current smoking at baseline (yes)	1.21 (0.96–1.52)	0.1103	1.14 (0.85–1.53)	0.3814
Aspartate aminotransferase at baseline (IU/L)	1.02 (1.00–1.03)	0.0261	1.01 (0.99–1.04)	0.3016
Alanine aminotransferase at baseline (IU/L)	1.01 (1.01–1.02)	0.0004	1.00 (1.00–1.02)	0.5427
HbA1c at baseline (%)	0.81 (0.71–0.93)	0.0036	0.89 (0.75–1.06)	0.1767
Log triglycerides at baseline	1.03 (1.00–1.07)	0.0495	1.01 (0.97–1.05)	0.7276
Amount of weight change after the date of NAFLD remission (kg)	1.27 (1.22–1.31)	< 0.0001	1.29 (1.24–1.34)	< 0.0001
Regular exercise at the last visit (yes)	0.61 (0.48–0.78)	< 0.0001	0.67 (0.55–0.89)	0.0053
Quitting smoking after the date of NAFLD remission (yes)	1.59 (1.02–2.48)	0.0393	1.31 (0.75–2.28)	0.3444
Follow-up duration (year)	1.06 (1.03–1.08)	< 0.0001	1.09 (1.06–1.12)	< 0.0001

**Figure 4:**
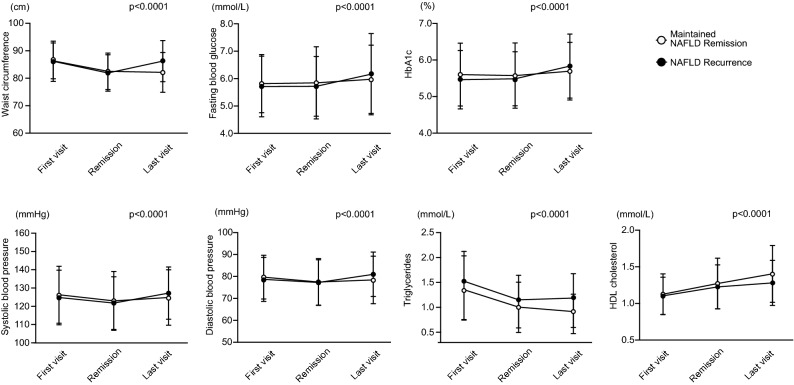
Changes in metabolic syndrome parameters. Data are shown as mean ± SD or median (25th, 75th). The p values were calculated using two-way repeated-measures analysis of variance. HbA1c, hemoglobin A1c; HDL, high-density lipoprotein; NAFLD, nonalcoholic fatty liver disease.

We also assessed the changes in metabolic syndrome parameters. The mean waist circumference at the last visit were 82.2 ± 6.8 cm and 86.3 ± 7.2 cm in the maintained remission and recurrence groups, respectively. The mean fasting blood glucose level remained low (5.98 ± 1.25 mmol/L) in the maintained NAFLD remission group and was higher (6.16 ± 1.48 mmol/L) in the recurrence group. The values of waist circumference, fasting blood glucose, HbA1c, systolic blood pressure, diastolic blood pressure, and serum triglycerides showed greater increases after remission in the recurrence group than in the maintained remission group. In addition, the mean serum HDL cholesterol level at the last visit was lower in the recurrence group than in the maintained remission group. Two-way repeated-measures analysis of variance revealed a significant interaction between time and groups on metabolic syndrome parameters (Fig. 4). This result indicated that NAFLD recurrence occurred simultaneously with worsening of metabolic syndrome parameters.

We compared metabolic syndrome parameters at the last visit and amount of weight change after NAFLD remission between the non-exercise and exercise groups of men (Table 3). Mean waist circumference was lower in the exercise group than the non-exercise group at the last visit (84.9 ± 7.2 cm vs. 82.7 ± 7.2 cm, *p* < 0.0001). The exercise group had lower serum triglyceride level (*p* = 0.0013) and higher HDL cholesterol level (*p* = 0.0002) at the last visit. The amount of weight change after NAFLD remission was lower in the exercise group than the non-exercise group (+ 1.6 ± 4.6 kg vs. + 0.6 ± 4.7 kg, *p* = 0.0006).

**Table 3:** Comparison of metabolic syndrome parameters at the last visit and amount of weight change after NAFLD remission between the non-exercise group and exercise group in men.

	ALL n = 1247	Non-exercise group (participants who do not exercise regularly at the last visit) n = 877	Exercise group (participants who exercise regularly at the last visit) n = 370	*p*
Waist circumference (cm)	84.3 (7.3)	84.9 (7.2)	82.7 (7.2)	< 0.0001
Fasting plasma glucose (mmol/L)	6.1 (1.4)	6.1 (1.4)	6.0 (1.3)	0.3168
HbA1c (%)	5.8 (0.8)	5.8 (0.9)	5.7 (0.8)	0.3681
Systolic blood pressure (mmHg)	126.0 (14.8)	125.7 (14.6)	126.5 (15.3)	0.3902
Diastolic blood pressure (mmHg)	79.7 (10.6)	79.8 (10.5)	79.3 (10.8)	0.4617
Triglycerides (mmol/L)	1.0 (0.7–1.5)	1.1 (0.7–1.5)	1.0 (0.7–1.3)	0.0013
HDL cholesterol (mmol/L)	1.3 (0.4)	1.3 (0.3)	1.4 (0.4)	0.0002
Amount of weight change after the date of NAFLD remission (kg)	+ 1.3 (4.7)	+ 1.6 (4.6)	+ 0.6 (4.7)	0.0006

**Figure 5:**
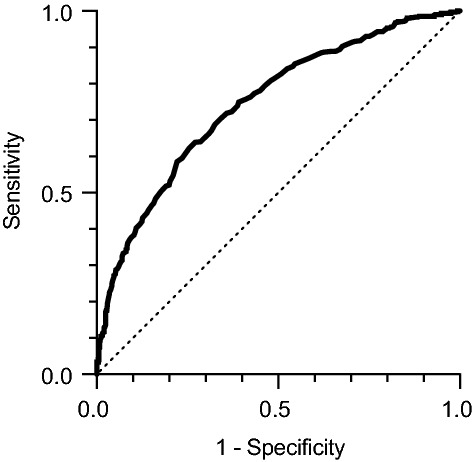
A receiver-operating characteristic (ROC) curve for body weight change after nonalcoholic fatty liver disease (NAFLD) remission to identify NAFLD recurrence (area under the ROC curve = 0.75, *p* < 0.0001). The cutoff value of weight change was + 1.5 kg (sensitivity 0.64, specificity 0.73).

A receiver operating characteristic (ROC) curve analysis demonstrated that the weight change after NAFLD remission was able to predict the development of NAFLD recurrence reasonably well (area under the ROC curve = 0.75, *p* < 0.0001) (Fig. 5). The cutoff value of weight change that best identified patients with NAFLD recurrence was + 1.5 kg (sensitivity, 0.64; specificity, 0.73).

The cohort study in women was designed as shown in the flow diagram in Supplementary Fig. S1. Among 20,671 women who were registered in NAGALA between May 1994 and Dec 2018, the number of those with fatty liver at the first visit was 1878. We excluded participants whose alcohol consumption was ≥ 20 g/day and those who had a known liver disease including viral hepatitis. We sorted out the participants whose fatty liver disappeared before the last visit. Finally, 264 participants were included in the female study. Among the 264 female participants who achieved NAFLD remission during the observation period, 143 (54.2%) remained in remission until the last visit and 121 (45.8%) showed recurrence at the last visit (Supplementary Fig. S1).

The clinical and laboratory characteristics at baseline in women are summarized in Supplementary Table S1. At baseline, the NAFLD recurrence group had a lower mean age, lower proportion of Fibrosis-4 (FIB-4) index (> 1.30 and < 2.67). The factors associated with NAFLD recurrence were assessed by univariable and multivariable logistic regression (Supplementary Table S2). The covariables included the characteristics at baseline that showed a significant intergroup difference: body weight change, follow-up period, age > 50 years (surrogate of menopausal state) at the last visit, exercise habits at the last visit, and quitting smoking after NAFLD remission. Multivariable logistic regression analysis showed that amount of weight change after the date of NAFLD remission (in 1 kg increments; aOR, 1.25; 95% CI, 1.15–1.35; *p* < 0.0001) and age > 50 years at the last visit (aOR, 4.01; 95% CI, 1.35–11.93; *p* = 0.0126) were independently associated with NAFLD recurrence.

## Discussion

In the present study, 49.0% of the male participants experienced recurrence after NAFLD remission. This result indicated that many patients develop NAFLD recurrence despite achieving remission once through lifestyle changes. However, as the importance of maintaining NAFLD remission has been rather overlooked, little is known about the crucial factors related to NAFLD recurrence. This study provides new insight into the management of NAFLD to prevent relapse and its associated complications.

Of note, there were no significant differences between the maintained NAFLD remission and recurrence group in body weight or BMI at baseline (Table 1). Our study found that weight regain after NAFLD remission was strongly and independently associated with NAFLD recurrence (Fig. 2, Table 2). The area under the ROC curve represents the predictive ability of weight change for NAFLD recurrence (Fig. 5). The closer the value is to 1.0, the higher its predictive power. The area under the ROC curve was 0.75, which suggests that the predictive power of weight change had moderate accuracy. A weight change of + 1.5 kg after NAFLD remission (sensitivity, 0.64; specificity, 0.73) well identified patients with NAFLD recurrence. It suggested that a weight regain of + 1.5 kg or more would lead NAFLD recurrence and be a good indicator to avoid body weight rebound and maintain NAFLD remission.

As previous reports showed the impact of weight gain in the development of NAFLD^21,22^, our result indicated that weight regain was related to its recurrence. Although body composition was not evaluated in this study, we examined the correlation between the change in body weight and the change in waist circumference after NAFLD remission. They were positively well correlated (R^2^ = 0.7005, *p* < 0.0001). This suggested that weight regain was associated with increasing amounts of visceral fat. NAFLD is defined by the accumulation of triglycerides in the liver. The sources of fatty acids that are required for the generation of hepatic triglycerides are principally derived from adipose tissue^23^. With increasing adipose tissue mass, specifically with the accumulation of dysfunctional and insulin-resistant adipocytes, release of fatty acids to the liver provides the substrate for and stimulus to hepatic lipid accumulation^24^.

Weight regain after successful weight loss is a major problem for many individuals. On average, more than half of the lost weight is regained within 2 years^25^. A previous study implied that weight loss–induced variations in cellular stress, extracellular matrix remodeling, inflammatory responses, adipokine secretion, and lipolysis are associated with the amount of weight that is regained after a successful weight loss^26^.

Our results also demonstrated that regular exercise at least once a week is independently important to prevent NAFLD recurrence (Fig. 3, Table 2). The comparison of exercise and non-exercise groups in the present study revealed that exercisers regained less weight and showed smaller mean waist circumference, lower serum triglyceride level and higher serum HDL cholesterol level at the last visit (Table 3). Previous studies demonstrated clear evidence of the benefits of exercise therapy for reducing liver fat^27^ and that the initiation of exercise is independently associated with NAFLD remission in men^22^. Consistent with the impact of exercise on NAFLD remission reported by previous studies, we revealed that the initiation or continuation of regular exercise is also important to preventing NAFLD recurrence. The mechanism for the direct hepatic benefit of regular exercise is inferred from rodent data. Regular exercise in rodents attenuates hepatic steatosis by reducing the lipogenic enzymes acetyl-coenzyme A carboxylase and fatty acid synthase^28^. Conversely, sedentary behavior in rats is marked by rapid reductions in hepatic fatty acid oxidation and mitochondrial enzyme activity (citrate synthase, β-hydroxyacyl-coA dehydrogenase, and cytochrome c oxidase), upregulation of fatty acid synthesis (increases in fatty acid synthase, stearoyl-coA desaturase-1, and sterol regulatory element binding protein 1c)^29^ and elevation of malonyl-CoA, which inhibits fatty acid oxidation^30^.

The present study’s findings indicated that quitting smoking is associated with NAFLD recurrence (Table 2). Although smoking cessation is known to decrease the risk of cardiovascular events^31^ and decrease the all-cause mortality rate^32^, quitting smoking carries a certain risk for weight gain^33^, which is associated with NAFLD recurrence. Hu et al. demonstrated that smoking cessation accompanied by weight gain is associated with an increased short-term risk of type 2 diabetes but does not mitigate the benefits in terms of reducing cardiovascular and all-cause mortality^34^. Smoking cessation should be recommended to all smokers to reduce mortality from all causes; however, the risk of weight gain and NAFLD recurrence after cessation should be considered.

Metabolic syndrome includes visceral obesity, insulin resistance, alterations in glucose and lipid metabolism, and increased blood pressure^35^. By investigating the changes in metabolic syndrome parameters^36^ in each group, we demonstrated that the values of waist circumference, fasting blood glucose, HbA1c, systolic blood pressure, diastolic blood pressure, and serum triglycerides were more elevated, while the mean HDL cholesterol level was lower in the NAFLD recurrence group than in the maintained remission group at the last visit (Fig. 4). Our findings are in line with the physiopathology of NAFLD, which has a mutual and bi-directional association with metabolic syndrome^4^. Therapies to prevent NAFLD recurrence are considered to reduce the risk of metabolic syndrome.

We also investigated the factors associated with NAFLD recurrence in women (Supplementary Tables S1, S2). Of all female patients, 9.1% were diagnosed with NAFLD at the first visit (Supplementary Fig. S1), a much lower proportion than that in the men (28.6% of all male participants). This finding was compatible with the fact that previous studies consistently indicated that the overall prevalence of NAFLD was higher in men than in women^37,38^. We also performed multivariate analysis including the covariable of age > 50 years as menopausal state at the last visit. Of note, the result showed that an age > 50 years at the last visit was independently associated with NAFLD recurrence (aOR, 4.01; 95% CI, 1.35–11.93; *p* = 0.0126), a finding in line with previous reports that mentioned NAFLD occurs at a higher rate in women after menopause, suggesting that estrogen is protective^20^. Although the analyses in women included a small population, we found that a weight change after remission was significantly related to NAFLD recurrence as in the male study. However, exercise habits at the last visit were not a significant factor in women.

Our study had several limitations. First, we categorized participants who regularly engaged in any kind of sport activity at least once a week as regular exercisers, whereas the exercise recommendation for health is generally 150 min of moderate-intensity physical activities plus resistance exercise twice a week by many organizations including the World Health Organization. Thus, it is important to evaluate physical activity mode, frequency, duration, and intensity. In a previous study, the hazard ratio for all-cause mortality was 0.66 (95% CI, 0.62–0.72) in insufficiently active participants who reported one to two exercise sessions per week versus inactive participants^39^. This suggests that any exercise performed more than once a week, which we defined as regular exercise in this study, contributes to health improvement.

Second, we did not assess the participants’ dietary habits. Weight gain is strongly affected by food intake variations^40^. It has also been suggested that having a tendency toward disinhibition or food addiction is associated with weight regain after a weight loss intervention^41^. Therefore, a survey of dietary and eating behaviors is needed to elucidate the independent role of each lifestyle modification.

Third, we performed a qualitative evaluation by ultrasonography to diagnose NAFLD but no quantitative evaluations. Evaluating semi-quantitative ultrasonographic indices is a useful and non-invasive way to investigate NAFLD severity, which varied among the participants^42^.

Next, there was an intergroup difference in the observation duration. However, we emphasized evaluating changes in lifestyle history, clinical parameters, and laboratory data between the time points. We also revealed that the intergroup differences in body weight changes remained significant after the degrees of change were divided by each observation duration. The mean amount of weight change between the date of NAFLD remission and the last visit divided by the duration in years was − 0.2 ± 1.7 and + 0.7 ± 1.1 kg/year (*p* < 0.0001) in the maintained NAFLD remission and recurrence groups, respectively.

A weight regain of 1.5 kg or more and lack of exercise are associated with NAFLD recurrence and worsening metabolic syndrome parameters in men. This study’s findings indicate the importance of weight loss maintenance and regular exercise for sustaining NAFLD remission.

## Methods

### Study design

The medical health check-ups including abdominal ultrasonography were performed in the Medical Health Checkup Center at Asahi University Hospital, Gifu, Japan. The details of the medical health check-up were previously described^5^. The results of the check-up were saved in a database after informed consent was obtained from the participants and their personally identifiable information was removed. This analysis was approved by the ethics committee of Asahi University Hospital (approval no. 2018-09-01) and performed in accordance with the Declaration of Helsinki. Informed consent was obtained via opt out on the website (https://www.hosp.asahi-u.ac.jp/shinryo/rinsyokenkyu/). Subjects who were unwilling to participate were excluded from the study.

### Data collection and measurement

We examined background factors including age, weight, BMI, waist circumference, family history of diabetes, blood pressure, lifestyle factors (smoking history, drinking history, regular exercise, coffee consumption, and sleep duration), and blood test results (aspartate aminotransferase, alanine aminotransferase, γ-glutamyl transferase, creatinine, fasting blood glucose, HbA1c, total cholesterol, triglyceride, HDL cholesterol, blood platelet count). The estimated glomerular filtration rate [eGFR] was calculated using the Japanese Society of Nephrology equation, as follows: eGFR = 194 × (serum creatinine) ^− 1.094^ × (age) ^− 0.287^ × 0.739 (if female) (mL/min/1.73 m^2^)^43^. FIB-4 index was calculated as (age × AST)/(blood platelet count × √ALT)^44^. We used a high cutoff value of FIB-4 index ≥ 2.67 and a low cutoff value of FIB-4 index ≤ 1.30^45^.

The diagnosis of fatty liver was made on the basis on the results of abdominal ultrasonography performed by trained technicians. All ultrasonographic images were stored as copies, and one gastroenterologist reviewed the images and made the diagnosis of fatty liver without referring to any other data. Of four known criteria (hepatorenal echo contrast, liver brightness, deep attenuation, and vascular blurring), the presence of both hepatorenal contrast and liver brightness was required to make a diagnosis of fatty liver^46^. To diagnose NAFLD, we excluded participants whose alcohol consumption was ≥ 30 g/day (≥ 210 g/week) for men and ≥ 20 g/day (≥ 140 g/week) for women^47^. We excluded known liver disease of drug-induced liver injury, cirrhosis, and fulminant hepatic failure according to what the participants stated in a questionnaire administered at the first visit. In addition, hepatitis B surface antigen and hepatitis C virus antibody was evaluated in the blood examination and we excluded the subjects when the result of either was positive at the first visit^48^.

### Standardized questionnaire about lifestyle factors

A standardized questionnaire was administered to all participants. Alcohol consumption habits were evaluated according to alcohol drink amount and type consumed per week during the past month and the mean ethanol intake per week was estimated. The participants were also categorized into three groups according to smoking status (never, former, and current). Exercise habits were asked in the questionnaires as follows: (1) Do you exercise regularly, occasionally, or not at all? and (2) How many times per week do you exercise? When the participants answered that they exercised regularly once or more a week, we categorized them into those who exercised regularly.

### Statistical analysis

Continuous values are presented as mean ± SD or median (interquartile range), while categorical values are presented as n (%). Student’s t test or the Wilcoxon signed-rank test were used to analyze the statistical differences in continuous variables. The analysis of categorical variables between the groups was performed using Pearson’s χ^2^ test. Two-way repeated-measures analysis of variance was performed to determine the existence of a significant interaction between time and group. We used univariable and multivariable logistic regression analyses to assess factors associated with NAFLD recurrence. An ROC curve analysis was conducted to evaluate the predictive ability of weight change for NAFLD recurrence and identify cutoff values of weight change that best identified participants with NAFLD recurrence. The cutoff value was at a point with high sensitivity and high specificity. All statistical analyses were performed using JMP Pro 15 software (https://www.jmp.com/en_us/software/predictive-analytics-software.html).

## Supplementary Information

Supplementary Information.

## References

[1] Younossi Z (2018). Global burden of NAFLD and NASH: Trends, predictions, risk factors and prevention. Nat. Rev. Gastroenterol. Hepatol..

[2] Ascha MS (2010). The incidence and risk factors of hepatocellular carcinoma in patients with nonalcoholic steatohepatitis. Hepatology.

[3] Negro F (2020). Natural history of NASH and HCC. Liver Int..

[4] Lonardo, A., Leoni, S., Alswat, K. A. & Fouad, Y. History of nonalcoholic fatty liver disease. *Int. J. Mol. Sci.* **21**, 5888. 10.3390/ijms21165888 (2020). 10.3390/ijms21165888 PMC7460697 32824337

[5] Hamaguchi M (2005). The metabolic syndrome as a predictor of nonalcoholic fatty liver disease. Ann. Intern. Med..

[6] Younossi ZM (2016). Global epidemiology of nonalcoholic fatty liver disease—Meta-analytic assessment of prevalence, incidence, and outcomes. Hepatology.

[7] Liu H, Lu HY (2014). Nonalcoholic fatty liver disease and cardiovascular disease. World J. Gastroenterol..

[8] Hamaguchi M (2007). Nonalcolholic fatty liver disease is a novel predictor cardiovascular disease. World J. Gastroenterol..

[9] Ortiz-Lopez C (2012). Prevalence of prediabetes and diabetes and metabolic profile of patients with nonalcoholic fatty liver disease (NAFLD). Diabetes Care.

[10] Fukuda T (2016). The impact of non-alcoholic fatty liver disease on incident type 2 diabetes mellitus in non-overweight individuals. Liver Int..

[11] Yamazaki H, Tsuboya T, Tsuji K, Dohke M, Maguchi H (2015). Independent association between improvement of nonalcoholic fatty liver disease and reduced incidence of type 2 diabetes. Diabetes Care.

[12] Musso G, Cassader M, Rosina F, Gambino R (2012). Impact of current treatments on liver disease, glucose metabolism and cardiovascular risk in non-alcoholic fatty liver disease (NAFLD): A systematic review and meta-analysis of randomised trials. Diabetologia.

[13] Koutoukidis DA (2019). Association of weight loss interventions with changes in biomarkers of nonalcoholic fatty liver disease: a systematic review and meta-analysis. JAMA Intern. Med..

[14] Promrat K (2010). Randomized controlled trial testing the effects of weight loss on nonalcoholic steatohepatitis. Hepatology.

[15] Vilar-Gomez E (2015). Weight loss through lifestyle modification significantly reduces features of nonalcoholic steatohepatitis. Gastroenterology.

[16] Hannah WN, Harrison SA (2016). Effect of weight loss, diet, exercise, and bariatric surgery on nonalcoholic fatty liver disease. Clin. Liver Dis..

[17] Osaka T (2018). Nonalcoholic fatty liver disease remission in men through regular exercise. J. Clin. Biochem. Nutr..

[18] Dureja P (2011). NAFLD recurrence in liver transplant recipients. Transplantation.

[19] Dumortier J (2010). Non-alcoholic fatty liver disease in liver transplant recipients: another story of ‘seed and soil’. Am. J. Gastroenterol..

[20] Lonardo A (2019). Sex differences in nonalcoholic fatty liver disease: state of the art and identification of research gaps. Hepatology.

[21] Yamada G (2020). Impact of body weight gain on the incidence of nonalcoholic fatty liver disease in nonobese Japanese individuals. Am. J. Gastroenterol..

[22] Yoshioka, N. *et al*. Effect of weight change and lifestyle modifications on the development or remission of nonalcoholic fatty liver disease: sex-specific analysis. *Sci. Rep.* **10**, 481. 10.1038/s41598-019-57369-9 (2020). 10.1038/s41598-019-57369-9 PMC6965633 31949229

[23] Tamura S, Shimomura I (2005). Contribution of adipose tissue and de novo lipogenesis to nonalcoholic fatty liver disease. J. Clin. Invest..

[24] Roden M, Shulman GI (2019). The integrative biology of type 2 diabetes. Nature.

[25] Anderson JW, Konz EC, Frederich RC, Wood CL (2001). Long-term weight-loss maintenance: a meta-analysis of US studies. Am. J. Clin. Nutr..

[26] van Baak MA, Mariman ECM (2019). Mechanisms of weight regain after weight loss—The role of adipose tissue. Nat. Rev. Endocrinol..

[27] Keating SE, Hackett DA, George J, Johnson NA (2012). Exercise and non-alcoholic fatty liver disease: A systematic review and meta-analysis. J. Hepatol..

[28] Rector RS (2008). Daily exercise increases hepatic fatty acid oxidation and prevents steatosis in Otsuka Long-Evans Tokushima Fatty rats. Am. J. Physiol. Gastrointest. Liver Physiol..

[29] Thyfault JP (2009). Rats selectively bred for low aerobic capacity have reduced hepatic mitochondrial oxidative capacity and susceptibility to hepatic steatosis and injury. J. Physiol..

[30] Rector RS (2008). Cessation of daily exercise dramatically alters precursors of hepatic steatosis in Otsuka Long-Evans Tokushima Fatty (OLETF) rats. J. Physiol..

[31] Mons, U. *et al*. Impact of smoking and smoking cessation on cardiovascular events and mortality among older adults: meta-analysis of Individual participant data from prospective cohort studies of the CHANCES consortium. *BMJ* **350**, h1551. 10.1136/bmj.h1551 (2015). 10.1136/bmj.h1551 PMC4413837 25896935

[32] Gellert C, Schöttker B, Brenner H (2012). Smoking and all-cause mortality in older people: Systematic review and meta-analysis. Arch. Intern. Med..

[33] Aubin, H. J., Farley, A., Lycett, D., Lahmek, P. & Aveyard, P. Weight gain in smokers after quitting cigarettes: Meta-analysis. *BMJ* **345**, e4439. 10.1136/bmj.e4439 (2012). 10.1136/bmj.e4439 PMC3393785 22782848

[34] Hu Y (2018). Smoking cessation, weight change, type 2 diabetes, and mortality. N. Engl. J. Med..

[35] Bosello O, Zamboni M (2000). Visceral obesity and metabolic syndrome. Obes. Rev..

[36] Grundy SM (2005). Diagnosis and management of the metabolic syndrome: An American Heart Association/National Heart, Lung, and Blood Institute scientific statement. Circulation.

[37] Lonardo, A. & Suzuki, A. Sexual dimorphism of NAFLD in adults. Focus on clinical aspects and implications for practice and translational research. *J. Clin. Med.* **9**, 1278. 10.3390/jcm9051278 (2020). 10.3390/jcm9051278 PMC7288212 32354182

[38] Wong VW-S (2012). Prevalence of non-alcoholic fatty liver disease and advanced fibrosis in Hong Kong Chinese: A population study using proton-magnetic resonance spectroscopy and transient elastography. Gut.

[39] O’Donovan G, Lee IM, Hamer M, Stamatakis E (2017). Association of ‘weekend warrior’ and other leisure time physical activity patterns with risks for all-cause, cardiovascular disease, and cancer mortality. JAMA Intern. Med..

[40] Mozaffarian D, Hao T, Rimm EB, Willett WC, Hu FB (2011). Changes in diet and lifestyle and long-term weight gain in women and men. N. Engl. J. Med..

[41] Sawamoto, R. *et al*. Predictors of successful long-term weight loss maintenance: a two-year follow-up. *Biopsychosoc. Med.* **11**, 14. 10.1186/s13030-017-0099-3 (2017). 10.1186/s13030-017-0099-3 PMC5460352 28592990

[42] Ballestri S (2020). Semi-quantitative ultrasonographic evaluation of NAFLD. Curr. Pharm. Des..

[43] Matsuo S (2009). Revised equations for estimated GFR from serum creatinine in Japan. Am. J. Kidney Dis..

[44] Sterling RK (2006). Development of a simple noninvasive index to predict significant fibrosis in patients with HIV/HCV coinfection. Hepatology.

[45] Shah AG (2009). Comparison of noninvasive markers of fibrosis in patients with nonalcoholic fatty liver disease. Clin. Gastroenterol. Hepatol..

[46] Hamaguchi M (2007). The severity of ultrasonographic findings in nonalcoholic fatty liver disease reflects the metabolic syndrome and visceral fat accumulation. Am. J. Gastroenterol..

[47] Chalasani N (2012). The diagnosis and management of non-alcoholic fatty liver disease: Practice guideline by the American Association for the Study of Liver Diseases, American College of Gastroenterology, and the American Gastroenterological Association. Hepatology.

[48] Chitturi S (2007). Non-alcoholic fatty liver disease in the Asia-Pacific region: Definitions and overview of proposed guidelines. J. Gastroenterol. Hepatol..

