# Supplementary material for: A weight regain of 1.5 kg or more and lack of exercise are associated with nonalcoholic fatty liver disease recurrence in men
Source: Sci Rep. 2021 Oct 7;11:19992. doi: 10.1038/s41598-021-99036-y (PMC8497533; doi:10.1038/s41598-021-99036-y)
Supplement: Supplementary file 1 — Supplementary Information. [file 41598_2021_99036_MOESM1_ESM.pdf]

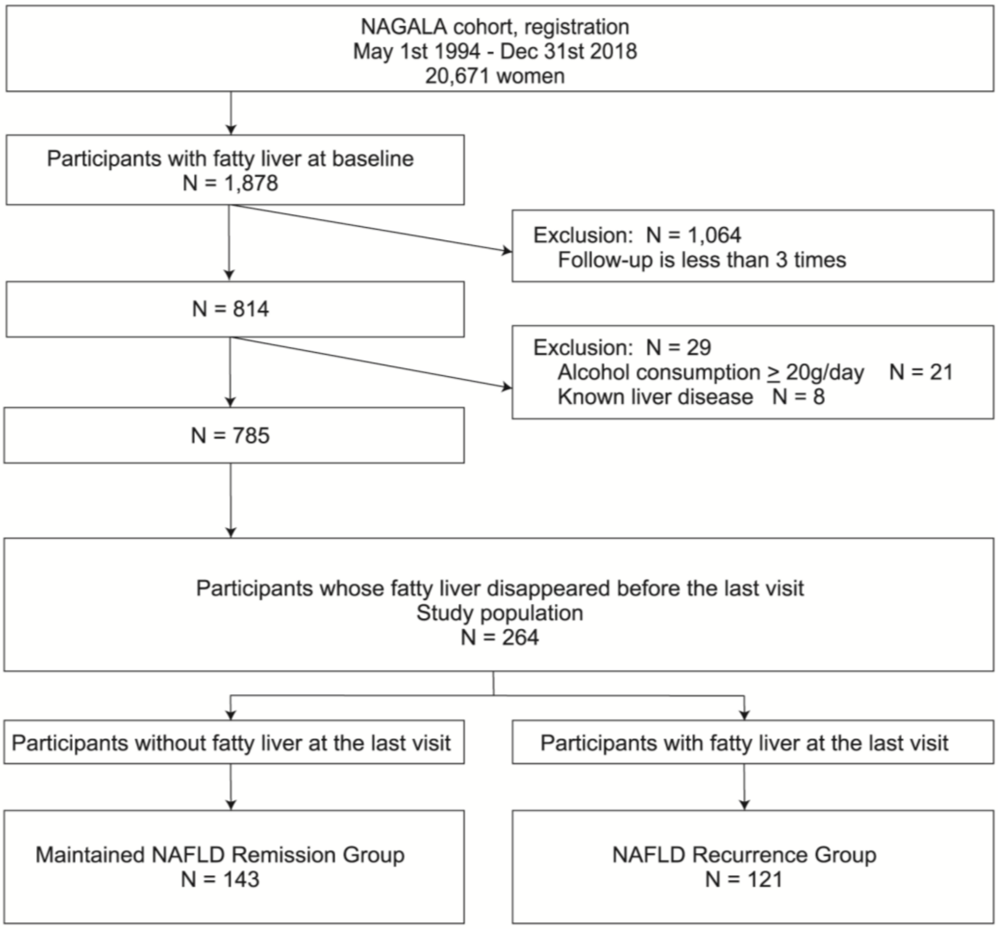

Supplementary Figure S1. Flow diagram of study population selection in women.

NAGALA, NAFLD in Gifu Area, Longitudinal Analysis; NAFLD, nonalcoholic fatty liver disease

Supplementary Table S1. Baseline characteristics of female study participants in the whole cohort and each group

Characteristics	ALL<br>n = 264	Maintained<br>NAFLD Remission<br>n = 143	NAFLD<br>Recurrence<br>n = 121	p
Age (year)	47.6 (9.5)	48.7 (10.2)	46.2 (8.4)	0.0319
Body weight (kg)	61.9 (9.2)	61.7 (9.2)	62.2 (9.3)	0.6691
Body mass index (kg/m^2)	25.2 (3.2)	25.1 (3.1)	25.4 (3.3)	0.5031
Parental history of diabetes (yes)	19 (7.2)	8 (5.6)	11 (9.1)	0.2734
Alcohol (Yes)	90 (36.4)	44 (32.4)	46 (41.4)	0.1398
Smoking (Non)	228 (86.4)	121 (84.6)	107 (88.4)	
(Past)	18 (6.8)	13 (9.1)	5 (4.1)	
(Current)	18 (6.8)	9 (6.3)	9 (7.4)	0.2725
Regular exercise (yes)	32 (12.3)	17 (12.1)	15 (12.5)	0.9133
Coffee intake (yes)	188 (71.2)	98 (68.5)	90 (74.4)	0.2957
Sleep duration per day (hour)	6.3 (1.0)	6.3 (1.1)	6.2 (1.0)	0.2119
Systolic blood pressure (mmHg)	125.6 (17.2)	126.7 (17.6)	124.4 (16.7)	0.2748
Diastolic blood pressure (mmHg)	77.5 (11.0)	77.8 (11.5)	77.1 (10.4)	0.6867

Aspartate aminotransferase (IU/L)	18.7 (7.1)	18.9 (7.9)	18.5 (6.2)	0.1771
Alanine aminotransferase (IU/L)	21.3 (12.1)	20.7 (13.3)	21.9 (10.7)	0.4538
γ-Glutamyltransferase (IU/L)	14.0 (11.0-20.8)	13.0(10.0-18.0)	15.0 (12.0-23.5)	0.0215
eGFR (ml/min/1.73m^2)	70.5 (15.2)	70.3 (14.7)	70.9 (5.9)	0.7480
Fasting plasma glucose (mmol/L)	5.7 (1.4)	5.6 (1.1)	5.8 (1.6)	0.2521
HbA1c (%)	5.6 (1.1)	5.6 (0.8)	5.7 (1.3)	0.4095
Total cholesterol (mmol/L)	5.7 (1.0)	5.8 (1.0)	5.7 (0.9)	0.2648
Triglycerides (mmol/L)	1.2 (0.9-1.6)	1.2 (0.9-1.5)	1.2 (0.9-1.6)	0.5690
HDL cholesterol (mmol/L)	1.4 (0.3)	1.4 (0.3)	1.3 (0.3)	0.1002
FIB-4 index (≤ 1.30)	234 (90.3)	120 (85.7)	114 (95.8)	
(>1.30 and < 2.67)	23 (8.9)	18 (12.9)	5 (4.2)	
(≥ 2.67)	2 (0.8)	2 (1.4)	0 (0.0)	0.0197

Supplementary Table S2. Odds ratio of NAFLD recurrence in women

	Unadjusted		Adjusted	
	**Odds ratio (95% confidence interval)**	**p**	**Odds ratio (95% confidence interval)**	**p**
Age at baseline (year)	0.97 (0.95-1.00)	0.0310	0.97 (0.91-1.02)	0.2330
Log γ-Glutamyltransferase at baseline	1.17 (0.97-1.40)	0.1015	1.21 (0.97-1.51)	0.0863
FIB-4 index at baseline	0.41 (0.20-0.81)	0.0102	0.66 (0.25-1.71)	0.3904
Amount of weight change after the date of NAFLD remission (kg)	1.23 (1.15-1.32)	<0.0001	1.25 (1.15-1.35)	<0.0001
Follow-up duration (year)	1.06 (1.03-1.08)	0.0189	1.05 (0.98-1.12)	0.5732
Age of > 50 years old at the last visit (yes)	1.17 (0.64-2.11)	0.6140	4.01 (1.35-11.93)	0.0126
Regular exercise at the last visit (yes)	0.90 (0.52-1.57)	0.7154	1.17 (0.61-2.25)	0.6351
Quitting smoking after the date of NAFLD remission (yes)	2.41 (0.43-13.39)	0.2987	0.82 (0.09-7.39)	0.8602
